# Self-reported depression and anxiety and healthcare professional interactions regarding smoking cessation and nicotine vaping: Findings from 2018 International Tobacco Control Four Country Smoking and Vaping (ITC 4CV) Survey

**DOI:** 10.18332/tpc/168288

**Published:** 2023-08-02

**Authors:** Bernadett E. Tildy, Ann McNeill, Katherine East, Shannon Gravely, Geoffrey T. Fong, K. Michael Cummings, Ron Borland, Gary C. K. Chan, Carmen C. W. Lim, Coral Gartner, Hua-Hie Yong, Leonie S. Brose

**Affiliations:** 1 Addictions Department, King’s College London, Addiction Sciences Building, London, United Kingdom; 2 SPECTRUM Consortium, London, United Kingdom; 3 Department of Psychology, University of Waterloo, Waterloo, Canada; 4 School of Public Health Sciences, University of Waterloo, Waterloo, Canada; 5 Ontario Institute for Cancer Research, Toronto, Canada; 6 Department of Psychiatry and Behavioral Sciences, Medical University of South Carolina, Charleston, United States; 7 School of Psychological Sciences, University of Melbourne, Melbourne, Victoria, Australia; 8 National Centre for Youth Substance Use Research, Faculty of Health and Behavioral Sciences, University of Queensland, Queensland, Australia; 9 School of Psychology, Faculty of Health and Behavioral Sciences, University of Queensland, Queensland, Australia; 10 NHMRC Centre of Research Excellence on Achieving the Tobacco Endgame, Faculty of Medicine, School of Public Health, University of Queensland, Herston, Australia; 11 School of Psychology, Deakin University, Geelong, Victoria, Australia

**Keywords:** depression, anxiety, mental health, smoking cessation, cessation advice, cessation support, quit smoking, vaping products, e-cigarette, health professional discussions

## Abstract

**INTRODUCTION:**

People with mental health conditions are disproportionately affected by smoking-related diseases and death. The aim of this study was to assess whether health professional (HP) interactions regarding smoking cessation and nicotine vaping products (NVPs) differ by mental health condition.

**METHODS:**

The cross-sectional 2018 International Tobacco Control Four Country (Australia, Canada, England, United States) Smoking and Vaping Survey data included 11040 adults currently smoking or recently quit. Adjusted weighted logistic regressions examined associations between mental health (self-reported current depression and/or anxiety) and visiting a HP in last 18 months; receiving advice to quit smoking; discussing NVPs with a HP; and receiving a recommendation to use NVPs.

**RESULTS:**

Overall, 16.1% self-reported depression and anxiety, 7.6% depression only, and 6.6% anxiety only. Compared with respondents with no depression/anxiety, those with depression (84.7%, AOR=2.65; 95% CI: 2.17–3.27), anxiety (82.2%, AOR=2.08; 95% CI: 1.70–2.57), and depression and anxiety (87.6%, AOR=3.74; 95% CI: 3.19–4.40) were more likely to have visited a HP. Among those who had visited a HP, 47.9% received advice to quit smoking, which was more likely among respondents with depression (AOR=1.58; 95% CI: 1.34–1.86), and NVP discussions were more likely among those with depression and anxiety (AOR=1.63; 95% CI: 1.29–2.06). Of the 6.1% who discussed NVPs, 33.5% received a recommendation to use them, with no difference by mental health.

**CONCLUSIONS:**

People with anxiety and/or depression who smoke were more likely to visit a HP than those without, but only those with depression were more likely to receive cessation advice, and only those with depression and anxiety were more likely to discuss NVPs. There are missed opportunities for HPs to deliver cessation advice. NVP discussions and receiving a positive recommendation to use them were rare overall.

## INTRODUCTION

Smoking is a leading preventable cause of illness and premature death in the United Kingdom (UK) and worldwide^1^. Smoking prevalence is considerably higher in disadvantaged groups, including people with mental health conditions ^2-5^. For example, in England in 2014, among those with a current common mental health condition, smoking prevalence was 34.1%, compared to 19.6% in people without^4^. In the United States (US), among those who reported any past-year mental illness in 2019, past-month cigarette smoking was 28.2%, compared to 15.8% in people without past-year mental illness^6^. People with mental health conditions are more likely to smoke heavily, and be highly dependent on cigarettes^4^. Smoking is a significant contributor to the discrepancy in life expectancy between people with and without mental health conditions^2,7,8^; smoking cessation should improve physical and mental health^9^.

Most adults who smoke say they want to quit smoking^10,11^, including people with mental health conditions^5^. Approximately 40–50% of adults who smoke report making a quit attempt annually, but most quit attempts are made without evidence-based treatments and relapse to smoking^10,11^. Health professionals (HPs) can trigger patients’ interest in quitting^12^ and provide treatments to support quit attempts, which can markedly increase cessation rates^13^. However, research has shown that the rate at which HPs provide advice to quit smoking and offer cessation support/treatment is suboptimal, internationally^14,15^. Nicotine vaping products (NVPs) are substantially less harmful than smoking combustible tobacco^16^ and improve cessation rates compared to nicotine replacement therapy (NRT) and non-nicotine vaping products^17^. However, there are concerns due to uncertainty about the long-term health effects of NVPs and youth uptake of NVPs. Some experts recommend that HPs encourage the use of NVPs as another option for smoking cessation on par with medicinally licensed pharmacotherapies and behavioral support^18,19^.

Policy and guidelines around NVPs vary internationally^18^. Currently, in the UK, NVPs are widely available as consumer products and clinical guidelines recommend that NVPs are ‘accessible to adults who smoke’^20^. In Australia, the sale of NVPs is prohibited unless on prescription from a licensed HP – clinical guidelines recommend NVPs for those ‘who have tried to achieve smoking cessation with first-line therapy but failed’^21^. In Canada, NVPs are widely available in various retail locations, but clinical guidelines do not include NVPs in the list of recommended smoking cessation treatment options^22^. In the US, historically NVPs were widely available on the open market, but only some tobacco-flavored brands have received market approval since 2021^23^. NVPs are not recommended in US clinical guidelines – ‘recommend that clinicians direct patients who use tobacco to other tobacco cessation interventions with proven effectiveness and established safety’^24^.

HPs rarely discuss NVPs with patients who smoke: in 2016 among people who smoked who visited a HP, only 6.8% of survey respondents from Australia, Canada, England, and the US reported their HP discussing NVPs with them^15^. A cohort study found that the prevalence of NVP discussions were low and remained relatively unchanged between 2016, 2018 and 2020^25^. Further, among respondents who discussed NVPs with HPs, only about one-third (37.8%) reported that their HP recommended that they use them^15^. The likelihood of receiving NVP recommendations from HPs in England was higher and increased significantly between 2016 and 2020, but did not change significantly in Australia, Canada or the US^25^.

To reduce smoking and narrow the inequalities in smoking prevalence that exist between people with and without mental health conditions, HPs needs to do more to assist those who smoke to quit – such as, increased guidance/encouragement for cessation and advising on harm reduction approaches (switching from smoking to using NVPs)^26,27^. One study^28^, using UK electronic health record data collected between 2009 and 2010, found that the annual mean number of consultations for patients who smoke and have a mental health condition was higher than for those without a mental health condition; however, the proportion of consultations in which cessation advice was recorded was lower for people with a mental health condition, compared to those without. Research into discussions and recommendations to use NVPs is sparse. One study^15^, using 2016 survey data from Australia, Canada, England and the US, found no difference in the proportion of people who smoke with and without self-reported current diagnosis/treatment of depression or anxiety who had discussions with a HP about NVPs; but fewer people who smoke with anxiety were recommended to use an NVP from their HPs, compared to people who smoke without anxiety.

In our study, we build on these findings, focusing on comparing respondents with and without depression and/or anxiety, as these are two of the most common mental health conditions globally^29^ but receive less attention compared to serious mental health illness^30^. Using cross-sectional 2018 International Tobacco Control Four Country Smoking and Vaping (ITC 4CV) Survey data from Australia, Canada, England and the US, this study investigated whether there were differences between those with and without a current diagnosis/treatment for depression and/or anxiety in: 1) visiting a HP; 2) receiving advice to quit smoking from a HP; 3) their HP discussing NVPs; and 4) receiving a positive recommendation to use NVPs from a HP. We also aimed to investigate if the association between depression and/or anxiety and each outcome varied by country.

## METHODS

### Data source and sample

This study used data from Wave 2 (March–June 2018) of the longitudinal ITC 4CV Survey, a cohort study of people who smoke, vape or those who recently quit smoking from Australia, Canada, England, and the US. Respondents (adults aged ≥18 years) were recruited using either probability-based sampling frames or non-probability opt-in sampling frames, or a combination of these methods, aiming to be representative of people who smoke, or vape at least weekly, in each country. Participants included those who were re-contacted from the previous wave and new participants who were recruited to address attrition and maintain sample size over time. Full methodological details are available elsewhere (https://itcproject.org/methods)^31^. This manuscript adhered to the STROBE guidelines.

**Figure 1:**
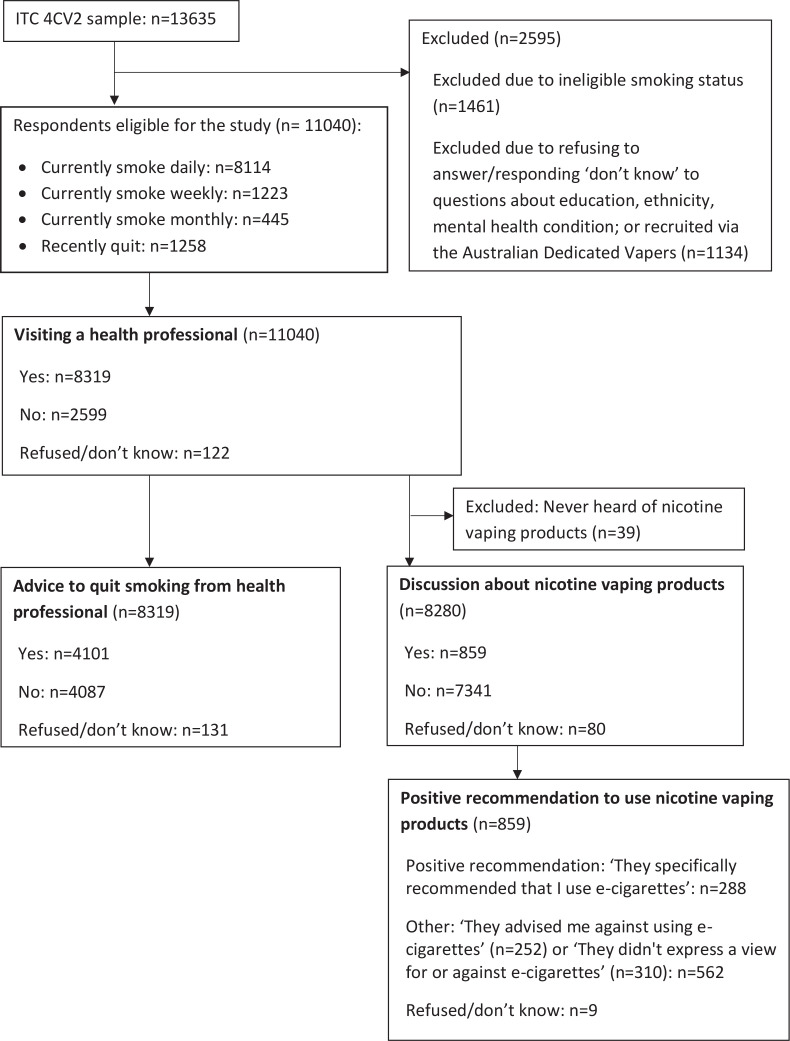
Flow diagram showing the inclusion/exclusion criteria to generate the study sample, from Wave 2 (2018) of the ITC 4CV Survey (unweighted frequencies)

The study sample consisted of 11040 adult respondents who were either currently smoking cigarettes (daily/weekly/monthly) or had recently quit (quit smoking in the last 18 months and had smoked >100 cigarettes in their lifetime), at the time of the 2018 survey (Figure 1).

A more detailed description of the variables is provided in the pre-registered analysis plan https://osf.io/y72cj^31^.

### Independent variable: mental health condition

The 2018 wave was the most recent ITC 4CV survey wave which contained survey questions about depression and anxiety (assessed with a single item measure, similar to past research)^15,32^. All respondents were asked: ‘Are you currently being treated for, or have you been diagnosed (current diagnosis) with, any of the following… [select all that apply]? Depression. Anxiety. …’. Response options: Selected/Not selected/Refused (excluded)/Don’t know (excluded). The answers were recoded into the mutually exclusive categories:

- No depression/anxiety: ‘Not selected’ to both depression and anxiety.
- Depression only: ‘Selected’ to depression but ‘Not selected’ to anxiety.
- Anxiety only: ‘Selected’ to anxiety but ‘Not selected’ to depression.
- Depression and anxiety: ‘Selected’ to both depression and anxiety.

### Outcome measures

*Visiting a HP*

All respondents were asked: *‘*In the last 18 months, have you visited a doctor or other health professional?’. Responses options were: yes, no, or refused to answer/don’t know (excluded).

*Advice to quit smoking from HP*

Respondents who indicated visiting a HP were asked: ‘On any visit to a doctor or health professional in the last 18 months, did you receive any advice to quit smoking?’. Responses options were: yes, no, or refused to answer/don’t know (excluded).

*Discussion about NVPs*

Respondents who indicated visiting a HP were asked: ‘On any visit to a doctor or health professional in the last 18 months, did the doctor or health professional talk to you about e-cigarettes?’. Responses options were: yes, no, or refused to answer/don’t know (excluded).

*Positive recommendation to use NVPs*

Respondents who indicated visiting a HP and indicated that their HP had discussed NVPs were asked: ‘What advice did the doctor or health professional give you about e-cigarettes?’. The response options were: ‘They specifically recommended that I use e-cigarettes’ (yes); ‘They advised me against using e-cigarettes’ (no); ‘They didn’t express a view for or against e-cigarettes’ (no); or refused to answer/don’t know (excluded).

*Covariates*

Covariates included: sex (male, female), age group (18–24, 25–39, 40–54, ≥55 years), country of residence (Australia, Canada, England, US), education level (low, moderate, high), ethnicity (Minority group, Majority group), annual household income level (low, moderate, high, no answer [valid response option]), cigarette smoking status (daily, non-daily [including weekly and monthly], former [quit smoking in the last 18 months and had smoked >100 cigarettes in their lifetime]), and problematic alcohol use (total score out of 12 based on the Alcohol Use Disorders Identification Test Consumption (AUDIT C)^33^ where: ≥5 points [yes], ≤4 points [no], or no answer [valid response option]).

Respondents who refused to answer or answered ‘don't know’ to the education level or ethnicity questions were excluded from the sample (Figure 1).

### Statistical analysis

Unweighted frequencies and weighted proportions were calculated. The sample was weighted using derived cross-sectional survey weights^31^ to account for the stratified sampling design (defined by geographical regions within each country). Respondents who refused to answer or responded ‘don’t know’ to a question related to the outcome measures were excluded from logistic regression analyses (Supplementary file Table 1). Three separate weighted logistic regression models were generated to investigate the relationship between mental health condition and the four outcomes: 1) visiting a HP; 2) receiving advice to quit smoking from a HP, among those who visited a HP; 3) their HP discussing NVPs, among those who visited a HP; and 4) receiving a positive recommendation to use NVPs from a HP, among those who visited a HP and whose HP discussed NVPs. The weighted regression models were: Model 1, unadjusted model with mental health condition as the only independent variable; Model 2, adjusted for country, sex, age, education level, ethnicity, and income level; and Model 3, fully adjusted model, using Model 2 but additionally adjusted for cigarette smoking status and problematic alcohol use. To assess whether the association between mental health condition and each outcome varies by country, for each outcome, a likelihood-ratio test assessed whether there was a significant difference between Model 3 and a new model (Model 4) which contained interaction terms between mental health condition and country.

Assumptions of logistic regression were met^34^. The analysis plan was pre-registered: https://osf.io/y72cj. Analyses were conducted using RStudio (version 4.0.3), regression models were generated using the *glm* command of the *mlogit* package. As the regressions were weighted, the ‘family=quasibinomial’ argument was used. Exact p-values and 95% (likelihood ratio-based^34^) confidence intervals (CIs) are reported. Results were adjusted for multiple comparisons, where the significance level, alpha, was evaluated at 0.0125 level, as per the Bonferroni correction (α=0.05/4 outcomes= 0.0125).

## RESULTS

### Sample characteristics

The unweighted analytical sample included 11040 respondents (Table 1). The weighted sample was 54.2% male, and respondents were more likely to be in the majority ethnic group (White) and aged ≥40 years. Most of the respondents were residing in England (38.6%), followed by Canada (27.8%), then the US (21.1%), and then Australia (12.5%). The most common cigarette smoking status was current ‘daily’ (77.7%). The ‘non-daily’ smoking category (11.8%) was made up of 8.4% who currently smoked weekly, and 3.4% who currently smoked monthly. People who recently quit smoking comprised 10.5% of respondents. The majority of respondents had a moderate education level (47.7%), moderate annual household income level (33.9%), and did not have problematic alcohol use (62.9%). Slightly less than one-third of the respondents had self-reported depression and/or anxiety (30.3%), 7.6% had depression only, 6.6% had anxiety only, and 16.1% had both depression and anxiety.

**Table 1:** Mental health condition and covariates by study sample and healthcare professional interactions regarding smoking cessation and nicotine vaping, cross-sectional ITC 4CV Survey, 2018 (N=11040)

*Variable*	*Study sample (N=11040) n (%)*	*Visiting a health professional (N=11040) n (%)*	*Advice to quit smoking from health professional (N=8319) n (%)*	*Discussion about nicotine vaping products (N=8280) n (%)*	*Positive recommendation to use nicotine vaping products (N=859) n (%)*
**Total**	11040 (100)	8319^a^ (74.6)^b^	4101^a^ (47.9)^b^	859^a^ (6.1)^b^	288^a^ (33.5)^b^
**Mental health status**					
No depression or anxiety	7393 (69.7)	5279 (69.8)	2550 (47.2)	459 (5.4)	150 (31.3)
Depression only	918 (7.6)	763 (84.7)	437 (57.0)	110 (7.4)	42 (38.8)
Anxiety only	844 (6.6)	662 (82.2)	317 (44.1)	89 (6.9)	28 (37.1)
Depression and anxiety	1885 (16.1)	1615 (87.6)	797 (47.3)	201 (8.0)	68 (35.0)
**Country**					
Australia	1372 (12.5)	1222 (85.7)	650 (53.5)	52 (3.0)	12 (16.6)
Canada	3157 (27.8)	2473 (79.3)	1159 (45.5)	228 (5.2)	64 (35.2)
England	4217 (38.6)	2822 (67.1)	1242 (42.1)	389 (8.4)	166 (39.0)
US	2294 (21.1)	1802 (75.3)	1050 (56.8)	190 (6.0)	46 (24.8)
**Gender**					
Male	5372 (54.2)	3777 (69.1)	1940 (49.5)	488 (6.8)	170 (38.0)
Female	5668 (45.8)	4542 (81.0)	2161 (46.2)	371 (5.5)	118 (27.9)
**Age** (years)					
18–24	2167 (9.8)	1427 (66.2)	610 (36.4)	262 (8.6)	93 (34.6)
25–39	2406 (33.6)	1617 (67.0)	708 (42.5)	215 (6.7)	83 (44.5)
40–54	2872 (28.6)	2198 (76.5)	1088 (48.1)	187 (5.8)	58 (27.1)
≥55	3595 (28.0)	3077 (84.5)	1695 (55.7)	195 (5.3)	54 (26.4)
**Ethnicity**					
Minority group	1636 (13.2)	1168 (72.7)	603 (50.9)	190 (8.3)	66 (33.5)
Majority group	9404 (86.8)	7151 (74.9)	3498 (47.4)	669 (5.8)	222 (33.4)
**Education level**					
Low	3519 (31.1)	2616 (74.5)	1283 (51.9)	224 (5.2)	72 (26.0)
Moderate	4627 (47.7)	3543 (74.5)	1771 (47.6)	346 (6.5)	97 (33.4)
High	2894 (21.2)	2160 (75.0)	1047 (42.7)	289 (6.8)	119 (42)
**Income level**					
Low	3533 (31.0)	2725 (76.5)	1347 (49.2)	242 (5.4)	67 (29.5)
Moderate	3706 (33.9)	2673 (72.7)	1331 (47.9)	278 (6.1)	94 (32.8)
High	3249 (30.0)	2499 (75.3)	1239 (46.7)	308 (6.9)	118 (38.8)
No answer	552 (5.1)	422 (71.0)	184 (45.8)	31 (6.0)	9 (22.6)
**Cigarette smoking status**					
Daily	8114 (77.7)	6142 (74.7)	3252 (51.8)	611 (6.0)	227 (34.6)
Non-daily	1668 (11.8)	1143 (69.8)	455 (32.1)	181 (8.2)	48 (31.5)
Former	1258 (10.5)	1034 (78.7)	394 (35.5)	67 (5.5)	13 (27.0)
**Problematic alcohol use**					
No	6951 (62.9)	5451 (76.9)	2735 (48.9)	501 (5.7)	160 (28.8)
Yes	3669 (33.4)	2599 (71.3)	1263 (46.2)	340 (7.2)	120 (41.0)
No answer	420 (3.7)	269 (65.1)	103 (43.3)	18 (4.3)	8 (33.3)

a Unweighted frequency of respondents who responded ‘Yes’ to the outcome.

b Weighted proportion of respondents who responded ‘Yes’ to the outcome (refused to answer and don't know responses were excluded from the denominator).

### Visiting a HP

Most (74.6%) respondents reported visiting a HP in the last 18 months (Table 1).

In all three regression models, compared to respondents with no depression/anxiety, the odds of visiting a HP in the last 18 months were significantly higher for respondents with these mental health conditions (Table 2). In the fully adjusted model (Model 3), the odds of visiting a HP were significantly higher for respondents with depression alone (AOR=2.65; 95% CI: 2.17–3.27, p<0.001), anxiety alone (AOR=2.08; 95% CI: 1.70–2.57, p<0.001), and both depression and anxiety (AOR=3.74; 95% CI: 3.19–4.40, p<0.001), compared to respondents with no depression/anxiety (Table 2).

**Table 2:** Logistic regression models to assess the association between mental health condition and healthcare professional interactions regarding smoking cessation and nicotine vaping, cross-sectional ITC 4CV Survey, 2018

	*Model 1*			*Model 2*			*Model 3*		
	*OR*	*95% CI*	*p*	*AOR*	*95% CI*	*p*	*AOR*	*95% CI*	*p*
**Visiting a health professional** (N=11040)									
No depression/anxiety (Ref.)	1			1			1		
Depression only	2.40	1.98–2.93	**<0.001**	2.62	2.15–3.23	**<0.001**	2.65	2.17–3.27	**<0.001**
Anxiety only	2.00	1.64–2.44	**<0.001**	2.08	1.70–2.57	**<0.001**	2.08	1.70–2.57	**<0.001**
Depression and anxiety	3.08	2.65–3.58	**<0.001**	3.71	3.17–4.36	**<0.001**	3.74	3.19–4.40	**<0.001**
**Advice to quit smoking from health professional** (N =8319)									
No depression/anxiety (Ref.)	1			1			1		
Depression only	1.48	1.27–1.74	**<0.001**	1.58	1.34–1.86	**<0.001**	1.58	1.34–1.86	**<0.001**
Anxiety only	0.88	0.74–1.05	0.152	0.95	0.80–1.14	0.601	0.94	0.79–1.12	0.493
Depression and anxiety	1.00	0.90–1.12	0.951	1.15	1.02–1.30	0.022	1.14	1.01–1.29	0.031
**Discussion about nicotine va ping products** (N=8280)									
No depression/anxiety (Ref.)	1			1			1		
Depression only	1.40	1.02–1.88	0.032	1.44	1.04–1.95	0.023	1.44	1.04–1.95	0.023
Anxiety only	1.30	0.92–1.81	0.126	1.45	1.01–2.03	0.036	1.45	1.01–2.03	0.037
Depression and anxiety	1.52	1.22–1.89	**<0.001**	1.65	1.30–2.09	**<0.001**	1.63	1.29–2.06	**<0.001**
**Positive recommendation to use nicotine vaping products** (N=8 59)									
No depression/anxiety (Ref.)	1			1			1		
Depression only	1.39	0.87–2.21	0.166	1.39	0.83–2.30	0.204	1.36	0.81–2.26	0.240
Anxiety only	1.30	0.76–2.17	0.331	1.06	0.60–1.86	0.831	1.02	0.57–1.81	0.954
Depression and anxiety	1.18	0.83–1.67	0.343	1.28	0.86–1.90	0.218	1.27	0.85–1.89	0.240

Model 1: unadjusted model with mental health condition as the only independent variable. Model 2: model adjusted for country, sex, age, education level, ethnicity, and income level. Model 3: fully adjusted model adjusted for country, sex, age, education level, ethnicity, income level, cigarette smoking status, and problematic alcohol use. The p-values smaller than our Bonferroni correction adjusted p=0.0125 are indicated in bold. AOR: adjusted odds ratio.

### Advice to quit smoking from HP

Among respondents who reported visiting a HP in the last 18 months, less than half (47.9%) reported receiving advice to quit smoking (Table 1).

In all three models, the odds of reporting receiving advice to quit smoking from a HP were significantly higher for respondents with depression alone, compared to respondents with no depression/anxiety (Table 2). In the fully adjusted model, the odds of reporting receiving advice to quit smoking from a HP were 1.58 times higher (95% CI: 1.34–1.86, p<0.001) for respondents with depression alone, compared to respondents with no depression/anxiety (Table 2). There was no significant difference in the odds of receiving advice to quit smoking between respondents with anxiety alone, and those with both depression and anxiety, compared to respondents with no depression/anxiety in any of the three models (Table 2).

### Discussion about NVPs

Among respondents who reported visiting a HP in the last 18 months, 6.1% (n=859) reported that their HP discussed NVPs with them (Table 1).

In all three models, there was a statistically significant difference in the odds of reporting a discussion about NVPs between respondents with both depression and anxiety compared to respondents with no depression/anxiety (Table 2). In the fully adjusted model (Model 3), the odds of reporting that their HP discussed NVPs were 1.63 times higher (95% CI: 1.29–2.06, p<0.001) for respondents with both depression and anxiety, compared to respondents with no depression/anxiety (Table 2). There was no significant difference in the odds of reporting HP NVP discussions between respondents with anxiety alone, and those with depression alone, compared to respondents with no depression/anxiety in any of the three models (Table 2).

### Positive recommendation to use NVPs

Among respondents who reported visiting a HP in the last 18 months and reported that the HP discussed NVPs with them, one-third (33.5%, n=288) reported receiving a positive recommendation from their HP to use NVPs (Table 1).

We did not find a significant association between mental health condition and the odds of receiving a positive recommendation to use NVPs in any of the three regression models (Table 2); however, sample sizes were small, so findings should be treated with caution.

### Country differences

Likelihood-ratio tests indicated a significant difference between the model with and without the mental health condition × country interaction terms for the ‘visiting a HP’ (p=0.002) and ‘receiving advice to quit smoking’ (p=0.009) outcomes. When we examined the individual interaction terms for mental health condition × country for these outcomes, only the depression and anxiety × Canada individual interaction term for ‘visiting a HP’ (p=0.001) was significant at p<0.01 (Supplementary file Tables 2d and 2h). We did not investigate country differences further.

## DISCUSSION

Most (74.6%) respondents reported visiting a HP in the last 18 months; the odds were higher for those respondents who reported anxiety and/or depression, compared to those with no depression/anxiety. Less than half of respondents (47.9%) who visited a HP reported receiving advice to quit smoking, with higher odds for those with depression alone. Among respondents who visited a HP, only 6.1% of respondents reported that their HP discussed NVPs with them; those with both depression and anxiety had higher odds. Lastly, among respondents who visited a HP and discussion with HPs included NVPs, one-third of respondents (33.5%) reported receiving a positive recommendation to use them and the odds did not differ by mental health condition (but our sample size was small). We also found that there may be a significant interaction between mental health condition and country regarding visiting a HP and receiving advice to quit smoking.

Our finding concerning HP visits was consistent with past research (which used 2009–2010 UK electronic health record data) which found that people with mental health conditions were more likely to visit HPs than those without^28^. Regarding cessation advice provision, past research found that the proportion of consultations in which cessation advice was recorded was lower for people with a mental health condition, compared to those without^28^. In our study, we found that people who had depression alone had higher odds of reporting being given advice to quit smoking from a HP compared to people with no depression/anxiety, with no significant differences for anxiety alone or having both conditions. However, as the number of consultations was not collected in the ITC survey, we could not explore whether this was due to a higher consultation rate among those with depression.

Regarding NVP discussions with HPs, consistent with existing studies which used survey data from Australia, Canada, England, and US from 2016^15^ and 2016–2020^25^, we found that a very low proportion of respondents who visited their HP reported their HP discussing NVPs with them. However, we found some evidence that those with both depression and anxiety had higher odds of their HPs discussing NVPs, compared to respondents with no depression/anxiety. The study investigating this in 2016^15^ found no difference by mental health status; however, they analyzed no anxiety versus anxiety and no depression versus depression. It may be that people who smoke who have both depression and anxiety were more likely to ask their doctor about NVPs or they may experience greater difficulty in quitting which may prompt their HP to mention NVPs as an alternative method to obtain nicotine. Further research is needed to substantiate this finding.

Lastly, unlike previous research – which found, using a previous wave (2016) of this survey, that people who smoke with anxiety were less likely to be recommended by their HP to use an NVP, compared to people who smoke without anxiety^15^ – we did not find an association between mental health condition and receiving a positive recommendation from a HP to use NVPs. Perhaps between 2016 and 2018, HPs increased the rate of recommendation of NVPs to their patients who have anxiety, so it was in line with their recommendation rate to patients who smoke without mental health conditions.

The consistency between older studies^14,15^ and our finding (using 2018 data) that less than half of all respondents received advice to quit smoking is notable because it indicates a lack of improvement in cessation advice provision in healthcare settings. It is promising that respondents with depression had a higher rate of receiving cessation advice (albeit only 57.2%), than respondents with no depression or anxiety, but this may be due to having a higher number of consultations in the last 18 months, as opposed to having a higher cessation intervention per visit rate^28^. Additionally, although those with anxiety either alone or with depression were also more likely to visit a HP, they were not more likely to receive cessation advice from their doctor (compared to those with no depression/anxiety), suggesting lower overall rates of intervention per visit among these groups. We advise that HPs increase the rate that they provide cessation advice and support to all their patients who smoke; this is particularly important for those who have mental health conditions to close the inequality gap of differential smoking rates^2-5^. Our finding that people who smoke with mental health conditions had higher odds of visiting a HP suggests that there are more opportunities for HPs to deliver cessation advice.

Our findings that only 6.1% of respondents who visited their HP reported their HP discussing NVPs with them, and only 2% received a positive recommendation to use them, are concerning given that NVPs have been found by Cochrane systematic reviews to be an effective quit method^17^. Furthermore, there was no association between receiving a positive recommendation by a HP to use NVPs and having anxiety or depression. It is especially important for people with anxiety/depression to be given accurate information about and access to NVPs, as various studies using surveys (e.g. 1993–2014 data from Great Britain)^4^ have found that people with mental health conditions are more likely to smoke heavily and be highly dependent on cigarettes, and are motivated to quit smoking (e.g. 2016–2017 data from England)^5^, but are less likely to succeed (e.g. 2016–2017 data from England^5,35^, 2016 data from Australia, Canada, England, and the US^36^).

To summarize, the main implications of this study are that there are missed opportunities for HPs to deliver cessation advice and to discuss NVPs in an evidence-based way with people who smoke with anxiety and/or depression. Given the higher smoking rates among people with mental health conditions^2-5^, to reduce the resultant health inequalities, HPs should increase the rate that they provide cessation advice and support per visit among people with mental health conditions. Also, although HPs should always consider the potential risks and benefits of recommending certain treatments, given that evidence suggests that using NVPs is substantially less harmful than smoking combustible tobacco^16^ and that NVPs have been shown to be a more efficacious smoking cessation aid than NRT^17^, HPs should at least discuss NVPs with their patients who smoke (with and without mental health conditions) when advising them about cessation options. This is particularly important given that currently effective licensed medications for smoking cessation (varenicline and bupropion) have been limited since 2021 and 2022.

### Future research

Future research could explore reasons behind why HPs provide differing care regarding smoking cessation to people with mental health conditions, and investigate if other forms of cessation support that HPs recommend to people who smoke (such as licensed cessation aids) differ by mental health status. Also, the effect of other mental health conditions should be investigated. To further investigate country effects, we recommend stratification by country, but a larger sample size will be required.

### Strengths and limitations

The strength of our cross-sectional study is that it used data from large population-based samples of people who smoke from four countries. However, there are some limitations. The study relies on self-reported measures which were not verified with health records, or other external measures, and may be subject to recall and other biases. It is not possible to know when a respondent was first diagnosed with depression and/or anxiety and the question used was not intended as a diagnostic tool. The sample size for some of our analyses was small.

## CONCLUSIONS

Using cross-sectional 2018 ITC Four Country (Australia, Canada, England, US) Survey data, this study found that people with anxiety and/or depression who smoke were more likely to visit a HP, but only people with depression alone were more likely to receive cessation advice, and only people with both depression and anxiety were more likely to discuss NVPs with their HP. Receiving a positive recommendation to use NVPs did not differ by mental health condition and few respondents received positive recommendations overall. More people who smoke should be given smoking cessation advice and information about effective smoking cessation support (including NVPs) to increase the likelihood of smoking cessation.

## Data Availability

In each country participating in the international Tobacco Control Policy Evaluation (ITC) Project, the data are jointly owned by the lead researcher(s) in that country and the ITC Project at the University of Waterloo. Data from the ITC Project are available to approved researchers 2 years after the date of issuance of cleaned data sets by the ITC Data Management Centre. Researchers interested in using ITC data are required to apply for approval by submitting an International Tobacco Control Data Repository (ITCDR) request application and subsequently to sign an ITCDR Data Usage Agreement. The criteria for data usage approval and the contents of the Data Usage Agreement are described online (http://www.itcproject.org).
