# Supplementary material for: Self-reported depression and anxiety and healthcare professional interactions regarding smoking cessation and nicotine vaping: Findings from 2018 International Tobacco Control Four Country Smoking and Vaping (ITC 4CV) Survey
Source: Tob Prev Cessat. 2023 Aug 2;9:26. doi: 10.18332/tpc/168288 (PMC10391919; doi:10.18332/tpc/168288)
Supplement: Supplementary file 1 [file TPC-9-26-s1.pdf]

SUPPLEMENTARY TABLES

Self-reported depression and anxiety and healthcare professional interactions regarding smoking cessation and nicotine vaping: Findings from 2018 International Tobacco Control (ITC) Four Country Smoking and Vaping Survey

Supplementary table 1. Mental health condition and covariates by healthcare professional interactions regarding smoking cessation and nicotine vaping. Cross-sectional International Tobacco Control Four Country Smoking and Vaping (ITC 4CV) Survey, 2018.

**Variable**	**Categories**	**Visiting a health professional** (n*= 11040)			**Advice to quit smoking from health professional** (n*= 8319)			**Discussion about nicotine vaping products** (n*= 8280)			**Positive recommendation to use nicotine vaping products** (n*= 859)		
		**No**	**Yes**	**Refused/don't know**	**No**	**Yes**	**Refused/don't know**	**No**	**Yes**	**Refused/don't know**	**No**	**Yes**	**Refused/don't know**
**TOTAL**		2599[+] (25.2[++])	8319 (74)	122 (0.7)	4087 (51.2)	4101 (47)	131 (1.7)	7341 (93.1)	859 (6.1)	80 (0.8)	562 (65.1)	288 (32.7)	9 (2.1)
**Gender**	**Male**	1523 (30.6)	3777 (68.5)	72 (0.9)	1775 (49.5)	1940 (48.5)	62 (2)	3232 (92.3)	488 (6.7)	41 (1)	313 (60.1)	170 (36.9)	5 (2.9)
	**Female**	1076 (18.9)	4542 (80.5)	50 (0.6)	2312 (53)	2161 (45.5)	69 (1.5)	4109 (93.9)	371 (5.5)	39 (0.6)	249 (71.4)	118 (27.6)	4 (1.1)
**Age group (years)**	**18-24**	683 (33.2)	1427 (65)	57 (1.8)	794 (62.6)	610 (35.8)	23 (1.6)	1135 (90.2)	262 (8.5)	21 (1.2)	168 (65.4)	93 (34.6)	1 (0.1)
	**25-39**	757 (32.7)	1617 (66.5)	32 (0.8)	881 (56.1)	708 (41.5)	28 (2.3)	1373 (92.3)	215 (6.6)	22 (1.1)	129 (53.9)	83 (43.3)	3 (2.8)
	**40-54**	650 (23.3)	2198 (75.9)	24 (0.8)	1077 (51.2)	1088 (47.4)	33 (1.4)	1979 (93.4)	187 (5.8)	21 (0.8)	128 (72.3)	58 (26.8)	1 (0.9)
	**55 and up**	509 (15.4)	3077 (84.4)	9 (0.2)	1335 (43.6)	1695 (54.9)	47 (1.5)	2854 (94.4)	195 (5.3)	16 (0.4)	137 (71.1)	54 (25.4)	4 (3.5)
**Ethnicity**	**Minority group**	438 (27)	1168 (71.7)	30 (1.3)	547 (48.2)	603 (50)	18 (1.8)	952 (89.9)	190 (8.1)	20 (2)	124 (66.5)	66 (33.5)	0 (0)
	**Majority group**	2161 (25)	7151 (74.4)	92 (0.6)	3540 (51.7)	3498 (46.6)	113 (1.7)	6389 (93.6)	669 (5.8)	60 (0.6)	438 (64.9)	222 (32.6)	9 (2.5)
**Education**	**Low**	861 (25.3)	2616 (74)	42 (0.7)	1283 (47)	1283 (50.6)	50 (2.4)	2351 (93.9)	224 (5.2)	26 (0.9)	151 (74)	72 (26)	1 (0)
	**Moderate**	1034 (25.3)	3543 (73.8)	50 (0.8)	1723 (51.7)	1771 (46.9)	49 (1.4)	3148 (92.9)	346 (6.4)	35 (0.7)	244 (64.1)	97 (32.1)	5 (3.8)
	**High**	704 (24.9)	2160 (74.6)	30 (0.5)	1081 (56.3)	1047 (42)	32 (1.7)	1842 (92.3)	289 (6.8)	19 (0.9)	167 (57.5)	119 (41.6)	3 (1)
**Income**	**Low**	767 (23.3)	2725 (76)	41 (0.7)	1330 (50)	1347 (48.4)	48 (1.6)	2435 (93.6)	242 (5.4)	32 (1.1)	170 (67.8)	67 (28.4)	5 (3.8)
	**Moderate**	982 (27.1)	2673 (72)	51 (0.9)	1296 (50.8)	1331 (46.7)	46 (2.5)	2360 (93)	278 (6.1)	24 (0.9)	183 (66.7)	94 (32.5)	1 (0.8)
	**High**	728 (24.5)	2499 (74.8)	22 (0.7)	1229 (52.7)	1239 (46.1)	31 (1.2)	2161 (92.6)	308 (6.9)	21 (0.5)	187 (59.8)	118 (38)	3 (2.3)
	**No answer**	122 (28.7)	422 (70.5)	8 (0.8)	232 (53.7)	184 (45.4)	6 (0.8)	385 (93.5)	31 (6)	3 (0.5)	22 (77.4)	9 (22.6)	0 (0)
**Cigarette smoking status**	**Daily**	1895 (25.1)	6142 (74.3)	77 (0.5)	2797 (47.5)	3252 (51)	93 (1.6)	5451 (93.5)	611 (5.9)	49 (0.6)	380 (64.5)	227 (34.1)	4 (1.4)
	**Non-daily**	488 (29.5)	1143 (68.3)	37 (2.2)	671 (66.4)	455 (31.3)	17 (2.3)	939 (90.2)	181 (8)	19 (1.7)	132 (68.4)	48 (31.5)	1 (0.2)
	**Former**	216 (21.2)	1034 (78.2)	8 (0.6)	619 (63.1)	394 (34.7)	21 (2.3)	951 (93.3)	67 (5.4)	12 (1.2)	50 (65.2)	13 (24.1)	4 (10.7)
**Problematic alcohol use**	**No**	1458 (23)	5451 (76.5)	42 (0.5)	2633 (50.3)	2735 (48.1)	83 (1.7)	4865 (93.3)	501 (5.7)	58 (1)	336 (70.2)	160 (28.4)	5 (1.4)
	**Yes**	1009 (28.5)	2599 (70.6)	61 (0.9)	1298 (52.8)	1263 (45.3)	38 (1.8)	2229 (92.4)	340 (7.2)	19 (0.4)	216 (57)	120 (39.6)	4 (3.4)
	**No answer**	132 (34)	269 (63.3)	19 (2.8)	156 (55.2)	103 (42.2)	10 (2.7)	247 (95.3)	18 (4.3)	3 (0.4)	10 (66.7)	8 (33.3)	0 (0)
**Mental health status**	**No depression or anxiety**	2049 (30)	5279 (69.3)	65 (0.7)	2650 (51.9)	2550 (46.4)	79 (1.7)	4738 (93.7)	459 (5.3)	56 (1)	302 (67.2)	150 (30.6)	7 (2.2)
	**Depression only**	137 (15.2)	763 (84.2)	18 (0.6)	312 (42)	437 (55.6)	14 (2.4)	643 (92.2)	110 (7.3)	7 (0.5)	68 (61.2)	42 (38.8)	0 (0)
	**Anxiety only**	162 (17.6)	662 (80.8)	20 (1.6)	333 (55)	317 (43.4)	12 (1.7)	564 (92.9)	89 (6.9)	6 (0.3)	61 (62.9)	28 (37.1)	0 (0)
	**Depression and anxiety**	251 (12.3)	1615 (87)	19 (0.7)	792 (51.9)	797 (46.6)	26 (1.5)	1396 (91.5)	201 (7.9)	11 (0.5)	131 (62.8)	68 (33.8)	2 (3.5)
**Country**	**Australia**	145 (14.2)	1222 (85.4)	5 (0.4)	553 (45.3)	650 (52.2)	19 (2.4)	1155 (96.7)	52 (3)	7 (0.3)	40 (83.4)	12 (16.6)	0 (0)
	**Canada**	659 (20.6)	2473 (78.9)	25 (0.5)	1285 (54)	1159 (45)	29 (1)	2217 (94.1)	228 (5.1)	22 (0.8)	162 (63.8)	64 (34.6)	2 (1.6)
	**England**	1325 (32.6)	2822 (66.4)	70 (1)	1539 (57.1)	1242 (41.6)	41 (1.3)	2385 (90.9)	389 (8.4)	31 (0.7)	216 (58.7)	166 (37.6)	7 (3.6)
	**US**	470 (24.5)	1802 (74.8)	22 (0.8)	710 (41.9)	1050 (55.1)	42 (3)	1584 (92.7)	190 (5.9)	20 (1.4)	144 (75.2)	46 (24.8)	0 (0)

* n is unweighted frequency, total number of respondents who were asked this survey question

+ Unweighted frequency of respondents who responded to the outcome

++ Weighted proportion of respondents who responded to the outcome. Denominator is frequency of respondents who responded ‘Yes’ and ‘No’ to the outcome, including refused and don’t know responses

Supplementary Table 2. Odds ratios, with 95% CI for all independent variables and covariates for all of the logistic regression models, for each outcome measure.

- Model 1: unadjusted model with mental health condition as the only independent variable
- Model 2: model adjusted for country, sex, age, education, ethnicity, and income
- Model 3 (fully adjusted): adjusted for country, sex, age, education, ethnicity, and income, cigarette smoking status and problematic alcohol use
- Model 4 (country-differences): adjusted for country, gender, age, education, income, ethnicity, cigarette smoking status, problematic alcohol use, and mental health*country interaction term

RQ1: Visiting a health professional

Supplementary Table 2a

Model 1

**Variable**	**Category**	**beta**	**OR**	**Lower 95% CI**	**Upper 95% CI**	**p-value**
	(Intercept)	0.84	2.31	2.20	2.42	0.000
Mental health	No depression/anxiety (ref)		1.00			
	Depression only	0.88	2.40	1.98	2.93	0.000
	Anxiety only	0.69	2.00	1.64	2.44	0.000
	Depression and anxiety	1.12	3.08	2.65	3.58	0.000

Supplementary Table 2b

Model 2

**Variable**	**Category**	**beta**	**OR**	**Lower 95% CI**	**Upper 95% CI**	**p-value**
	(Intercept)	-0.63	0.53	0.42	0.67	0.000
Mental health	No depression/anxiety (ref)		1.00			
	Depression only	0.96	2.62	2.15	3.23	0.000
	Anxiety only	0.73	2.08	1.70	2.57	0.000
	Depression and anxiety	1.31	3.71	3.17	4.36	0.000
Gender	Male (ref)		1.00			
	Female	0.68	1.98	1.79	2.18	0.000
Age	18-24 (ref)		1.00			
	25-39	0.09	1.09	0.93	1.28	0.287
	40-54	0.67	1.96	1.66	2.31	0.000
	55 and up	1.35	3.84	3.22	4.58	0.000

Ethnicity	Minority group		1.00			
	Majority group	-0.07	0.94	0.81	1.07	0.339
Education	Low (ref)		1.00			
	Moderate	0.24	1.27	1.14	1.42	0.000
	High	0.29	1.33	1.16	1.53	0.000
Income	Low (ref)		1.00			
	Moderate	0.11	1.11	0.99	1.25	0.083
	High	0.23	1.26	1.11	1.43	0.000
	No answer	-0.09	0.91	0.73	1.14	0.418
Country	England (ref)		1.00			
	Australia	1.19	3.30	2.77	3.95	0.000
	Canada	0.65	1.91	1.69	2.15	0.000
	US	0.43	1.54	1.35	1.75	0.000

Supplementary Table 2c

Model 3 (fully adjusted)

Variable	Category	beta	OR	Lower 95% CI	Upper 95% CI	p-value
	(Intercept)	-0.57	0.57	0.45	0.72	0.000
Mental health	No depression/anxiety (ref)		1.00			
	Depression only	0.98	2.65	2.17	3.27	0.000
	Anxiety only	0.73	2.08	1.70	2.57	0.000
	Depression and anxiety	1.32	3.74	3.19	4.40	0.000
Gender	Male (ref)		1.00			
	Female	0.67	1.95	1.77	2.15	0.000
Age	18-24 (ref)		1.00			
	25-39	0.06	1.06	0.90	1.24	0.468
	40-54	0.66	1.93	1.63	2.29	0.000
	55 and up	1.32	3.75	3.14	4.49	0.000
Ethnicity	Minority group		1.00			
	Majority group	-0.07	0.93	0.81	1.07	0.305
Education	Low (ref)		1.00			
	Moderate	0.24	1.27	1.13	1.42	0.000
	High	0.27	1.31	1.14	1.50	0.000
Income	Low (ref)		1.00			
	Moderate	0.10	1.10	0.98	1.24	0.109

	High	0.22	1.25	1.10	1.42	0.001
	No answer	-0.08	0.92	0.74	1.15	0.471
Country	England (ref)		1.00			
	Australia	1.19	3.27	2.75	3.91	0.000
	Canada	0.63	1.88	1.67	2.12	0.000
	US	0.40	1.49	1.31	1.70	0.000
Problematic alcohol use (Y/N)	No		1.00			
	Yes	-0.08	0.93	0.84	1.02	0.133
	No answer	-0.37	0.69	0.55	0.87	0.002
Cigarette smoking status	Daily (ref)		1.00			
	Non-daily	-0.08	0.92	0.80	1.06	0.257
	Former	0.35	1.42	1.21	1.67	0.000

Supplementary Table 2d

Model 4 (country-differences)

Variable	Category	beta	OR	Lower 95% CI	Upper 95% CI	p-value
	(Intercept)	-0.61	0.54	0.43	0.69	0.000
Mental health	No depression/anxiety (ref)		1.00			
	Depression only	1.27	3.58	2.70	4.81	0.000
	Anxiety only	0.77	2.17	1.55	3.08	0.000
	Depression and anxiety	1.56	4.76	3.77	6.06	0.000
Country	England (ref)		1.00			
	Australia	1.26	3.53	2.90	4.32	0.000
	Canada	0.73	2.08	1.82	2.38	0.000
	US	0.47	1.60	1.38	1.85	0.000
Gender	Male (ref)		1.00			
	Female	0.67	1.96	1.78	2.16	0.000
Age	18-24 (ref)		1.00			
	25-39	0.06	1.06	0.90	1.24	0.484
	40-54	0.65	1.92	1.62	2.28	0.000
	55 and up	1.32	3.75	3.13	4.49	0.000
Ethnicity	Minority group		1.00			
	Majority group	-0.07	0.93	0.81	1.07	0.296
Education	Low (ref)		1.00			
	Moderate	0.23	1.26	1.13	1.41	0.000

	High	0.27	1.31	1.14	1.51	0.000
Income	Low (ref)		1.00			
	Moderate	0.10	1.11	0.98	1.25	0.100
	High	0.23	1.25	1.10	1.43	0.001
	No answer	-0.07	0.93	0.75	1.16	0.518
Problematic alcohol use (Y/N)	No		1.00			
	Yes	-0.08	0.92	0.84	1.02	0.123
	No answer	-0.38	0.68	0.54	0.86	0.001
Cigarette smoking status	Daily (ref)		1.00			
	Non-daily	-0.09	0.91	0.79	1.05	0.209
	Former	0.34	1.41	1.20	1.66	0.000
Mental health*country interaction term	No depression/anxiety*England (ref)		1.00			
	Depression only*Australia	-0.59	0.55	0.28	1.18	0.103
	Anxiety only*Australia	-0.65	0.52	0.27	1.05	0.060
	Depression and anxiety*Australia	0.08	1.09	0.58	2.21	0.802
	Depression only*Canada	-0.62	0.54	0.32	0.92	0.021
	Anxiety only*Canada	-0.11	0.89	0.54	1.49	0.659
	Depression and anxiety*Canada	-0.64	0.53	0.36	0.78	0.001
	Depression only*US	-0.63	0.53	0.31	0.91	0.020
	Anxiety only*US	0.24	1.27	0.72	2.27	0.412
	Depression and anxiety*US	-0.44	0.64	0.44	0.95	0.027

Likelihood-ratio test between Model 3 (fully adjusted) and Model 4 (country-differences): p=0.002

RQ2: Advice to quit smoking from health professional

Supplementary Table 2e

Model 1

**Variable**	**Category**	**beta**	**OR**	**Lower 95% CI**	**Upper 95% CI**	**p-value**
	(Intercept)	-0.11	0.89	0.85	0.94	0.000
Mental health	No depression/anxiety (ref)		1.00			
	Depression only	0.39	1.48	1.27	1.74	0.000
	Anxiety only	-0.13	0.88	0.74	1.05	0.152

	Depression and anxiety	0.00	1.00	0.90	1.12	0.951

Supplementary Table 2f

Model 2

**Variable**	**Category**	**beta**	**OR**	**Lower 95% CI**	**Upper 95% CI**	**p-value**
	(Intercept)	-0.57	0.57	0.45	0.72	0.000
Mental health	No depression/anxiety (ref)		1.00			
	Depression only	0.46	1.58	1.34	1.86	0.000
	Anxiety only	-0.05	0.95	0.80	1.14	0.601
	Depression and anxiety	0.14	1.15	1.02	1.30	0.022
Gender	Male (ref)		1.00			
	Female	-0.09	0.91	0.83	1.00	0.043
Age	18-24 (ref)		1.00			
	25-39	0.25	1.29	1.08	1.54	0.005
	40-54	0.49	1.63	1.37	1.96	0.000
	55 and up	0.80	2.23	1.87	2.68	0.000
Ethnicity	Minority group		1.00			
	Majority group	-0.22	0.81	0.70	0.92	0.002
Education	Low (ref)		1.00			
	Moderate	-0.02	0.98	0.88	1.09	0.737
	High	-0.25	0.78	0.69	0.89	0.000
Income	Low (ref)		1.00			
	Moderate	0.05	1.05	0.94	1.18	0.365
	High	0.00	1.00	0.89	1.13	0.986
	No answer	0.02	1.02	0.82	1.26	0.876
Country	England (ref)		1.00			
	Australia	0.46	1.58	1.37	1.83	0.000
	Canada	0.12	1.13	1.01	1.26	0.040
	US	0.55	1.73	1.52	1.96	0.000

Supplementary Table 2g

<u>Model 3 (fully adjusted)</u>

Variable	Category	beta	OR	Lower 95% CI	Upper 95% CI	p-value
	(Intercept)	-0.35	0.71	0.55	0.90	0.006
Mental health	No depression/anxiety (ref)		1.00			
	Depression only	0.45	1.58	1.34	1.86	0.000
	Anxiety only	-0.06	0.94	0.79	1.12	0.493
	Depression and anxiety	0.14	1.14	1.01	1.29	0.031
Gender	Male (ref)		1.00			
	Female	-0.11	0.89	0.81	0.98	0.016
Age	18-24 (ref)		1.00			
	25-39	0.16	1.17	0.98	1.41	0.090
	40-54	0.34	1.40	1.17	1.69	0.000
	55 and up	0.65	1.92	1.60	2.31	0.000
Ethnicity	Minority group		1.00			
	Majority group	-0.23	0.79	0.69	0.91	0.001
Education	Low (ref)		1.00			
	Moderate	0.03	1.03	0.92	1.14	0.627
	High	-0.14	0.87	0.76	0.99	0.039
Income	Low (ref)		1.00			
	Moderate	0.05	1.05	0.94	1.18	0.367
	High	0.04	1.04	0.92	1.18	0.515
	No answer	0.06	1.07	0.86	1.33	0.570
Country	England (ref)		1.00			
	Australia	0.46	1.58	1.36	1.82	0.000
	Canada	0.16	1.17	1.05	1.32	0.007
	US	0.60	1.82	1.60	2.08	0.000
Problematic alcohol use (Y/N)	No		1.00			
	Yes	0.00	1.00	0.90	1.10	0.939
	No answer	-0.20	0.82	0.63	1.06	0.129
Cigarette smoking status	Daily (ref)		1.00			
	Non-daily	-0.72	0.49	0.42	0.57	0.000
	Former	-0.67	0.51	0.44	0.60	0.000

Supplementary Table 2h

Model 4 (country-differences)

Variable	Category	beta	OR	Lower 95% CI	Upper 95% CI	p-value
	(Intercept)	-0.32	0.73	0.56	0.93	0.013
Mental health	No depression/anxiety (ref)		1.00			
	Depression only	0.59	1.81	1.41	2.32	0.000
	Anxiety only	-0.18	0.84	0.58	1.19	0.330
	Depression and anxiety	0.01	1.01	0.82	1.23	0.954
Country	England (ref)		1.00			
	Australia	0.31	1.37	1.14	1.64	0.001
	Canada	0.14	1.15	1.00	1.32	0.047
	US	0.64	1.90	1.62	2.23	0.000
Gender	Male (ref)		1.00			
	Female	-0.11	0.89	0.81	0.98	0.017
Age	18-24 (ref)		1.00			
	25-39	0.14	1.15	0.96	1.38	0.130
	40-54	0.33	1.39	1.16	1.67	0.000
	55 and up	0.64	1.90	1.58	2.29	0.000
Ethnicity	Minority group		1.00			
	Majority group	-0.22	0.80	0.70	0.92	0.002
Education	Low (ref)		1.00			
	Moderate	0.02	1.02	0.92	1.14	0.689
	High	-0.14	0.87	0.76	1.00	0.048
Income	Low (ref)		1.00			
	Moderate	0.05	1.05	0.94	1.18	0.408
	High	0.04	1.04	0.92	1.17	0.566
	No answer	0.05	1.05	0.84	1.31	0.667
Problematic alcohol use (Y/N)	No		1.00			
	Yes	0.00	1.00	0.91	1.11	0.968
	No answer	-0.20	0.82	0.63	1.06	0.131
Cigarette smoking status	Daily (ref)		1.00			
	Non-daily	-0.72	0.49	0.42	0.57	0.000
	Former	-0.67	0.51	0.44	0.60	0.000
Mental health*country interaction term	No depression/anxiety*England (ref)		1.00			

	Depression only*Australia	0.17	1.19	0.73	1.94	0.490
	Anxiety only*Australia	0.76	2.15	1.20	3.89	0.011
	Depression and anxiety*Australia	0.36	1.44	1.01	2.04	0.044
	Depression only*Canada	-0.30	0.74	0.49	1.12	0.150
	Anxiety only*Canada	0.16	1.17	0.74	1.87	0.508
	Depression and anxiety*Canada	0.18	1.20	0.89	1.62	0.235
	Depression only*US	-0.56	0.57	0.36	0.92	0.020
	Anxiety only*US	-0.21	0.81	0.49	1.35	0.420
	Depression and anxiety*US	0.12	1.13	0.82	1.55	0.468

Likelihood-ratio test between Model 3 (fully adjusted) and Model 4 (country-differences): p=0.009

RQ3: Discussion about nicotine vaping products

Supplementary Table 2i

<u>Model 1</u>

**Variable**	**Category**	**beta**	**OR**	**Lower 95% CI**	**Upper 95% CI**	**p-value**
	(Intercept)	-2.87	0.06	0.05	0.06	0.000
Mental health	No depression/anxiety (ref)		1.00			
	Depression only	0.33	1.40	1.02	1.88	0.032
	Anxiety only	0.27	1.30	0.92	1.81	0.126
	Depression and anxiety	0.42	1.52	1.22	1.89	0.000

Supplementary Table 2j

<u>Model 2</u>

**Variable**	**Category**	**beta**	**OR**	**Lower 95% CI**	**Upper 95% CI**	**p-value**
	(Intercept)	-1.99	0.14	0.09	0.21	0.000
Mental health	No depression/anxiety (ref)		1.00			
	Depression only	0.36	1.44	1.04	1.95	0.023
	Anxiety only	0.37	1.45	1.01	2.03	0.036
	Depression and anxiety	0.50	1.65	1.30	2.09	0.000
Gender	Male (ref)		1.00			

	Female	-0.31	0.73	0.61	0.88	0.001
Age	18-24 (ref)		1.00			
	25-39	-0.14	0.87	0.64	1.21	0.399
	40-54	-0.28	0.76	0.55	1.05	0.093
	55 and up	-0.29	0.75	0.54	1.05	0.092
Ethnicity	Minority group		1.00			
	Majority group	-0.39	0.68	0.53	0.87	0.002
Education	Low (ref)		1.00			
	Moderate	0.00	1.00	0.80	1.26	0.988
	High	0.09	1.09	0.83	1.44	0.531
Income	Low (ref)		1.00			
	Moderate	0.06	1.06	0.83	1.35	0.654
	High	0.32	1.38	1.07	1.77	0.012
	No answer	0.10	1.11	0.68	1.71	0.668
Country	England (ref)		1.00			
	Australia	-1.13	0.32	0.22	0.46	0.000
	Canada	-0.53	0.59	0.46	0.74	0.000
	US	-0.43	0.65	0.50	0.84	0.001

Supplementary Table 2k

<u>Model 3 (fully adjusted)</u>

**Variable**	**Category**	**beta**	**OR**	**Lower 95% CI**	**Upper 95% CI**	**p-value**
	(Intercept)	-2.06	0.13	0.08	0.20	0.000
Mental health	No depression/anxiety (ref)		1.00			
	Depression only	0.36	1.44	1.04	1.95	0.023
	Anxiety only	0.37	1.45	1.01	2.03	0.037
	Depression and anxiety	0.49	1.63	1.29	2.06	0.000
Gender	Male (ref)		1.00			
	Female	-0.29	0.75	0.62	0.91	0.004
Age	18-24 (ref)		1.00			
	25-39	-0.11	0.89	0.65	1.24	0.501
	40-54	-0.25	0.78	0.56	1.10	0.149
	55 and up	-0.24	0.78	0.56	1.11	0.163
Ethnicity	Minority group		1.00			
	Majority group	-0.41	0.67	0.52	0.86	0.002

Education	Low (ref)		1.00			
	Moderate	-0.01	0.99	0.79	1.25	0.938
	High	0.08	1.09	0.82	1.43	0.556
Income	Low (ref)		1.00			
	Moderate	0.05	1.05	0.82	1.34	0.698
	High	0.31	1.36	1.06	1.75	0.016
	No answer	0.12	1.12	0.70	1.75	0.615
Country	England (ref)		1.00			
	Australia	-1.11	0.33	0.22	0.47	0.000
	Canada	-0.54	0.58	0.46	0.74	0.000
	US	-0.41	0.67	0.51	0.86	0.002
Problematic alcohol use (Y/N)	No		1.00			
	Yes	0.14	1.15	0.94	1.39	0.180
	No answer	-0.40	0.67	0.34	1.19	0.210
Cigarette smoking status	Daily (ref)		1.00			
	Non-daily	0.16	1.18	0.89	1.54	0.240
	Former	-0.12	0.89	0.64	1.20	0.462

Supplementary Table 2l

Model 4 (country-differences)

**Variable**	**Category**	**beta**	**OR**	**Lower 95% CI**	**Upper 95% CI**	**p-value**
	(Intercept)	-2.10	0.12	0.08	0.19	0.000
Mental health	No depression/anxiety (ref)		1.00			
	Depression only	0.45	1.57	1.02	2.34	0.033
	Anxiety only	0.73	2.07	1.21	3.39	0.005
	Depression and anxiety	0.40	1.50	1.06	2.09	0.019
Country	England (ref)		1.00			
	Australia	-1.04	0.35	0.21	0.56	0.000
	Canada	-0.49	0.61	0.46	0.82	0.001
	US	-0.42	0.66	0.47	0.92	0.015
Gender	Male (ref)		1.00			
	Female	-0.29	0.75	0.62	0.91	0.004
Age	18-24 (ref)		1.00			
	25-39	-0.10	0.91	0.66	1.26	0.562
	40-54	-0.23	0.80	0.57	1.12	0.178

	55 and up	-0.22	0.80	0.57	1.14	0.207
Ethnicity	Minority group		1.00			
	Majority group	-0.40	0.67	0.52	0.87	0.002
Education	Low (ref)		1.00			
	Moderate	0.00	1.00	0.79	1.26	0.990
	High	0.10	1.11	0.84	1.46	0.481
Income	Low (ref)		1.00			
	Moderate	0.04	1.04	0.81	1.32	0.768
	High	0.30	1.34	1.05	1.73	0.021
	No answer	0.13	1.14	0.70	1.76	0.585
Problematic alcohol use (Y/N)	No		1.00			
	Yes	0.15	1.16	0.95	1.41	0.150
	No answer	-0.41	0.67	0.34	1.18	0.200
Cigarette smoking status	Daily (ref)		1.00			
	Non-daily	0.17	1.18	0.89	1.55	0.227
	Former	-0.12	0.88	0.64	1.19	0.434
Mental health*country interaction term	No depression/anxiety*England (ref)		1.00			
	Depression only*Australia	0.33	1.39	0.47	3.67	0.519
	Anxiety only*Australia	-1.47	0.23	0.01	1.22	0.159
	Depression and anxiety*Australia	-0.14	0.87	0.34	2.06	0.758
	Depression only*Canada	-0.36	0.70	0.27	1.59	0.417
	Anxiety only*Canada	-0.49	0.61	0.26	1.37	0.240
	Depression and anxiety*Canada	0.07	1.07	0.60	1.88	0.806
	Depression only*US	-0.40	0.67	0.24	1.63	0.404
	Anxiety only*US	-0.57	0.57	0.22	1.37	0.222
	Depression and anxiety*US	0.37	1.44	0.82	2.53	0.202

Likelihood-ratio test between Model 3 (fully adjusted) and Model 4 (country-differences): p=0.415

RQ4: Positive recommendation to use nicotine vaping products

Supplementary Table 2m

Model 1

Variable	Category	beta	OR	Lower 95% CI	Upper 95% CI	p-value
	(Intercept)	-0.78697	0.46	0.37	0.55	0.000
Mental health	No depression/anxiety (ref)		1.00			
	Depression only	0.329851	1.39	0.87	2.21	0.166
	Anxiety only	0.259387	1.30	0.76	2.17	0.331
	Depression and anxiety	0.167331	1.18	0.83	1.67	0.343

Supplementary Table 2n

Model 2

Variable	Category	beta	OR	Lower 95% CI	Upper 95% CI	p-value
	(Intercept)	-0.78584	0.46	0.22	0.92	0.029
Mental health	No depression/anxiety (ref)		1.00			
	Depression only	0.328175	1.39	0.83	2.30	0.204
	Anxiety only	0.06195	1.06	0.60	1.86	0.831
	Depression and anxiety	0.247724	1.28	0.86	1.90	0.218
Gender	Male (ref)		1.00			
	Female	-0.4939	0.61	0.45	0.83	0.002
Age	18-24 (ref)		1.00			
	25-39	0.354833	1.43	0.87	2.37	0.164
	40-54	-0.49003	0.61	0.36	1.05	0.073
	55 and up	-0.32982	0.72	0.42	1.24	0.233
Ethnicity	Minority group		1.00			
	Majority group	0.252891	1.29	0.83	2.01	0.259
Education	Low (ref)		1.00			
	Moderate	0.196878	1.22	0.82	1.82	0.334
	High	0.556871	1.75	1.10	2.78	0.019
Income	Low (ref)		1.00			
	Moderate	0.023276	1.02	0.68	1.54	0.911
	High	0.17508	1.19	0.78	1.82	0.417

	No answer	-0.31529	0.73	0.30	1.62	0.457
Country	England (ref)		1.00			
	Australia	-1.28757	0.28	0.12	0.56	0.001
	Canada	-0.04017	0.96	0.65	1.42	0.841
	US	-0.5582	0.57	0.37	0.89	0.013

Supplementary Table 2o

Model 3 (fully adjusted)

**Variable**	**Category**	**beta**	**OR**	**Lower 95% CI**	**Upper 95% CI**	**p-value**
	(Intercept)	-0.76783	0.46	0.22	0.98	0.045
Mental health	No depression/anxiety (ref)		1.00			
	Depression only	0.307861	1.36	0.81	2.26	0.240
	Anxiety only	0.020313	1.02	0.57	1.81	0.945
	Depression and anxiety	0.238358	1.27	0.85	1.89	0.240
Gender	Male (ref)		1.00			
	Female	-0.4655	0.63	0.45	0.87	0.005
Age	18-24 (ref)		1.00			
	25-39	0.250262	1.28	0.77	2.15	0.338
	40-54	-0.63619	0.53	0.30	0.92	0.024
	55 and up	-0.40505	0.67	0.38	1.17	0.159
Ethnicity	Minority group		1.00			
	Majority group	0.19366	1.21	0.78	1.90	0.393
Education	Low (ref)		1.00			
	Moderate	0.264145	1.30	0.87	1.96	0.201
	High	0.726933	2.07	1.28	3.36	0.003
Income	Low (ref)		1.00			
	Moderate	0.008834	1.01	0.67	1.53	0.966
	High	0.092974	1.10	0.71	1.69	0.674
	No answer	-0.41987	0.66	0.27	1.49	0.333
Country	England (ref)		1.00			
	Australia	-1.20143	0.30	0.13	0.62	0.002
	Canada	0.095251	1.10	0.73	1.65	0.645
	US	-0.45725	0.63	0.40	0.99	0.048
Problematic alcohol use (Y/N)	No		1.00			
	Yes	0.332198	1.39	0.98	1.97	0.061

	No answer	0.23702	1.27	0.41	3.55	0.661
Cigarette smoking status	Daily (ref)		1.00			
	Non-daily	-0.49388	0.61	0.38	0.96	0.038
	Former	-0.45376	0.64	0.34	1.13	0.133

Supplementary Table 2p

Model 4 (country-differences)

**Variable**	**Category**	**beta**	**OR**	**Lower 95% CI**	**Upper 95% CI**	**p-value**
	(Intercept)	-0.68906	0.50	0.23	1.08	0.081
Mental health	No depression/anxiety (ref)		1.00			
	Depression only	-0.12952	0.88	0.44	1.72	0.709
	Anxiety only	0.358144	1.43	0.63	3.28	0.392
	Depression and anxiety	0.288494	1.33	0.76	2.34	0.316
Country	England (ref)		1.00			
	Australia	-2.09746	0.12	0.03	0.40	0.002
	Canada	0.207044	1.23	0.73	2.05	0.430
	US	-0.5784	0.56	0.30	1.02	0.064
Gender	Male (ref)		1.00			
	Female	-0.54273	0.58	0.41	0.81	0.002
Age	18-24 (ref)		1.00			
	25-39	0.229897	1.26	0.75	2.14	0.392
	40-54	-0.58543	0.56	0.32	0.99	0.044
	55 and up	-0.40161	0.67	0.38	1.20	0.173
Ethnicity	Minority group		1.00			
	Majority group	0.151895	1.16	0.74	1.85	0.514
Education	Low (ref)		1.00			
	Moderate	0.256897	1.29	0.85	1.98	0.232
	High	0.70506	2.02	1.23	3.35	0.006
Income	Low (ref)		1.00			
	Moderate	0.038565	1.04	0.68	1.60	0.859
	High	0.085482	1.09	0.70	1.71	0.708
	No answer	-0.47473	0.62	0.25	1.44	0.285
Problematic alcohol use (Y/N)	No		1.00			
	Yes	0.360392	1.43	1.00	2.06	0.050
	No answer	0.26229	1.30	0.41	3.77	0.638

Cigarette smoking status	Daily (ref)		1.00			
	Non-daily	-0.55277	0.58	0.35	0.92	0.025
	Former	-0.45563	0.63	0.34	1.16	0.149
Mental health*country interaction term	No depression/anxiety*England (ref)		1.00			
	Depression only*Australia	2.801618	16.47	2.47	136.18	0.005
	Anxiety only*Australia	-10.774	0.00	NA	2276945316589920000000000	0.979
	Depression and anxiety*Australia	0.719362	2.05	0.15	19.32	0.538
	Depression only*Canada	0.933002	2.54	0.61	10.56	0.196
	Anxiety only*Canada	-1.54585	0.21	0.04	0.93	0.052
	Depression and anxiety*Canada	-0.34615	0.71	0.27	1.80	0.471
	Depression only*US	0.381836	1.46	0.22	7.51	0.663
	Anxiety only*US	0.195571	1.22	0.24	5.52	0.804
	Depression and anxiety*US	0.118814	1.13	0.41	3.09	0.818

Likelihood-ratio test between Model 3 (fully adjusted) and Model 4 (country-differences): p=0.064

© 2023 Tildy B. E. et al.
